# Keratinocyte derived extracellular vesicles mediated crosstalk between epidermis and dermis in UVB-induced skin inflammation

**DOI:** 10.1186/s12964-024-01839-9

**Published:** 2024-09-30

**Authors:** Yubin Li, Avital Baniel, DeAnna Diaz, Mariko Ogawa-Momohara, Cristina Ricco, Ahmed Eldaboush, Muhammad Bashir, Meena Sharma, Ming-Lin Liu, Victoria P. Werth

**Affiliations:** 1 Corporal Michael J. Crescenz Veterans Affairs Medical Center, Philadelphia, PA USA; 2 Department of Dermatology, School of Medicine, University of Pennsylvania, 3400 Civic Center Boulevard, Philadelphia, PA 19104 USA

## Abstract

**Background and rationale:**

Ultraviolet-B (UVB) light induces dermal inflammation, although it is mostly absorbed in the epidermis. Recent reports suggest extracellular vesicles (EVs) act as a mediator of photodamage signaling. Melatonin is reported to be a protective factor against UV-induced damage. We hypothesized that EVs derived from UVB-irradiated keratinocytes might trigger proinflammatory responses in dermal cells and tested whether melatonin can ameliorate UVB-induced inflammation.

**Methods:**

We used UVB-irradiated HaCaT cells, primary keratinocytes and STING knock-out mice to model production of EVs under photodamaging conditions and performed immunoblotting and ELISA to measure their effect on dermal macrophages.

**Results:**

UVB-irradiated keratinocytes produce an increased number of EVs that contain higher concentrations of DNA and protein compared with controls. KC-derived EVs (KEVs) induced a STING- and inflammasome-mediated proinflammatory response in macrophages in vitro, and a pronounced inflammatory infiltrate in mouse dermis in vivo. Melatonin ameliorated KEVs inflammatory effect both in vitro and in vivo.

**Conclusions:**

This data suggests EVs are mediators in a crosstalk that takes place between keratinocytes and their neighboring cells as a result of photodamage. Further studies exploring EVs induced by damaging doses of UVB, and their impact on other cells will provide insight into photodamage and may help develop targeted therapeutic approaches.

**Supplementary Information:**

The online version contains supplementary material available at 10.1186/s12964-024-01839-9.

## Introduction

The profound impact of ultraviolet radiation (UVR) on the skin is well known, yet the mechanism underlying the transformation of UV light absorbed in the epidermis into immune signaling affecting the dermis and systemic circulation is less understood. In general, low or sub-erythemogenic doses exert an immunosuppressive effect that predominantly affects the adaptive immune system [35]. Yet UVR is mostly known for its immunostimulatory effect that is the cause of many deleterious effects of UVR on the skin, including sunburn, skin cancer, photodermatoses, photo-aggravation of inflammatory diseases and ultimately autoimmune connective tissue diseases. These harmful effects are attributed mainly to the innate immune system [5].

The major cause of photodamage and photoaging is irradiation in the ultraviolet B (UVB) range, 290-320 nm [1]. Because the epidermal layers of skin absorb almost all UVB, with just 5-10% reaching the dermis [1], a major question in photobiology is how UVB irradiation can generate dermal and systemic effects.

Extracellular vesicles (EVs) may suggest one explanation to this question. EVs are small lipid bilayer membrane structures that are released by normal, diseased, and transformed cells in vitro and in vivo [47]. EVs carry nucleic acids (DNA, mRNAs, non-coding RNAs), lipids including peroxidized lipids, and proteins to mediate cell-cell communication [13]. Many of the molecules carried by EVs act as damage-associated molecular patterns (DAMPs) that activate innate immunity [14, 49]. Abnormal EVs have been implicated in the pathogenesis of photosensitive autoimmune diseases such as lupus erythematosus, psoriasis, and dermatomyositis [41], although their cells of origin and pathologic significance remain unclear [3]. Previous studies have confirmed that EVs are present in human skin [38]. Crosstalk via EVs has been observed in vitro amongst several types of skin cells, including keratinocytes (KCs), melanocytes, human dermal fibroblasts, dermal papilla cells, and microvascular endothelial cells [29]. In a mouse model of psoriasis, KEVs activated neutrophils and enhanced skin inflammation. Treatment of psoriatic mice with GW4869, a known inhibitor of EV generation, significantly alleviated the skin lesions by reducing epidermal thickness and numbers of infiltrating cells [22]. Because KCs constitute the majority of skin cells, KEVs released from UVB-irradiated KCs may also alter the pathophysiological behavior of neighboring keratocytes and other skin cells [36]. A recent study demonstrated the uptake of DNA adducts carried by UVB-irradiated KEVs by bystander keratinocytes [9]. Another study demonstrated the ability of a post-translational modification in Dsg2 to promote tumor progression in squamous cell carcinoma by modulation of EV biogenesis and cargo [16] .

A growing body of evidence implicates the stimulator of interferon genes (STING) signaling pathway in EV-mediated proinflammatory responses [12, 24, 33, 40]. The mechanism underlying STING activation is related to the nucleic acid cargo of the EVs. Many types of EVs contain both nuclear DNA and mitochondrial DNA [27]. The DNA, inside or attached to the surface of these EVs, can directly stimulate cytosolic cyclic GMP-AMP synthase (cGAS) within target immune cells. During states of acute or chronic inflammation, cellular stress may promote EV generation, vascular permeability, extracellular release of nucDNA and mtDNA, and migration of resident inflammatory cells to initiate pathological cytokines production [31]. UVB exposure triggers significant cutaneous and systemic Type-1 IFN responses that are highly dependent on upregulation of the STING pathway [37]. We sought to investigate whether KEVs have a role in UVB-triggered inflammation through the STING pathway.

Melatonin is recognized as an important protective factor in many different cell types [34, 39]. Melatonin can also inhibit inflammasome activation, thereby acting as an anti-inflammatory agent [15].

We reasoned that UVB radiation provokes keratinocytes in the epidermis to release biologically active EVs that then leave the epidermis to act on immune cells in the dermis to confer a proinflammatory effect. We demonstrate this effect is mediated by the cGAS-STING pathway and is ameliorated by melatonin.

## Results

### UVB irradiation induced extracellular vesicles release from keratinocytes

**Fig. 1:**
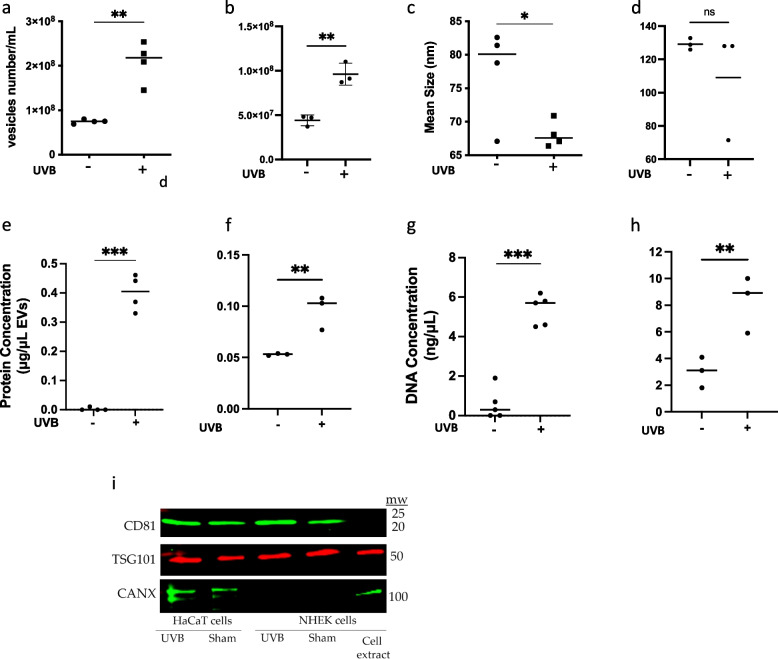
Characterization of keratinocyte-derived EVs (KEVs). HaCaT keratinocytes were plated in 35-mm wells (5x10^5^/mL, 2mL/well) and then irradiated with 0 (-) or 75mJ/cm^2^ of UVB (+), NHEKs were plated in 35-mm wells (2.7x10^6^/mL, 2mL/well) and then irradiated with 0 (-) or 30 mJ/cm^2^ of UVB (+). 15 hours after UVB irradiation, culture supernatants were collected for isolation of KEVs. Total number of KEVs derived from both UVB-irradiated HaCaT cells (**a**) and primary keratinocytes (**b**) is higher than in sham-irradiated cells. Average size of KEVs derived from sham-irradiated HaCaT keratinocytes is larger than UVB-irradiated HaCaT keratinocytes (**c**), in NHEKs no significant size difference was detected (**d**). Protein content of KEVs derived from sham- and UVB-irradiated HaCaT (**e**) and NHEK (**f**) cells. DNA content of KEVs derived from sham- and UVB-irradiated HaCaT (**g**) and NHEK (**h**) cells. Western blot demonstrates enriched EV markers CD81 and TSG101 in representative samples. CANX was not detected in NHEKs but was detected in HaCaT cells (**i**). Data represent means±SDs. *p<0.05, **p<0.01, ***p<0.001 between groups as indicated

UVB irradiation (75 mJ/cm^2^) of HaCaT keratinocytes provoked the release of 5-fold more extracellular vesicles than from sham-irradiated cells (Fig. 1a) with a smaller mean size (Fig. 1c). UVB-irradiated keratinocyte-derived EVs contained more protein (Fig. 1e) and DNA (Fig. 1g) than KEVs from sham-irradiated keratinocytes. We obtained similar results for primary cells. Irradiated neonatal human epidermal keratinocytes (NHEK) provoked the release of more EVs (Fig. 1b) and contained more protein (Fig. 1f) and DNA (Fig. 1h). After UVB EV size was not significantly different (Fig. 1d). EV extracts of both HaCaT and NHEK cells demonstrated enriched CD81 and TSG101 on western blot. Calnexin was negative in NHEKs but detected in small amount in HaCaT cells. This protein may be detected in microvesicles as previously described [18]. UVB exposure did not significantly affect cell viability. Fifteen hours after exposure death rates were 11% and 5% for HaCaT and primary cells respectively.

### UVB irradiation released KEVs triggered STING-mediated proinflammatory responses in PBMCs and macrophages

**Fig. 2:**
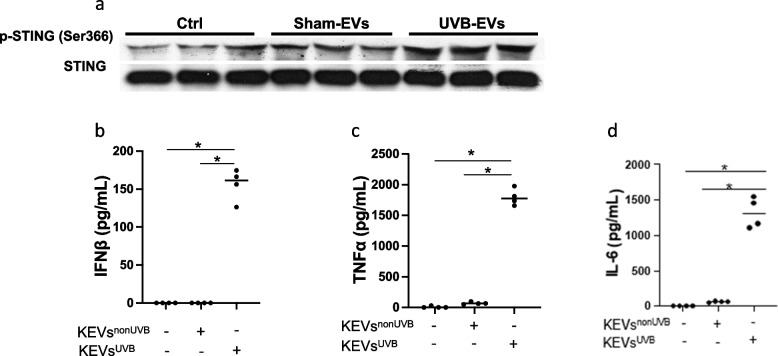
UVB-irradiated keratinocyte-derived EVs (KEVs-UVB) triggered more STING phosphorylation and proinflammatory cytokines in primary human PBMCs and macrophages. UVB-irradiated KEVs triggered more STING phosphorylation than non-UVB-irradiated keratinocyte-derived EVs in PBMCs (**a**). UVB-irradiated keratinocyte-derived EVs triggered more IFNβ (**b**), TNFα (**c**) and IL-6 (**d**) in macrophages. Data represent mean±SD. *p<0.05, between groups as indicated

EVs derived from same volume of conditioned medium of non-UVB or UVB irradiated HaCaT keratinocytes were used to stimulate human peripheral blood mononuclear cells (PBMCs) and primary human macrophages. UVB-irradiated KEVs induced more STING phosphorylation in PBMCs (Fig. 2a) but not in macrophages (data not shown). Nevertheless, increased proinflammatory cytokine production was demonstrated in primary human macrophages (Fig. 2b-d).

**Fig. 3:**
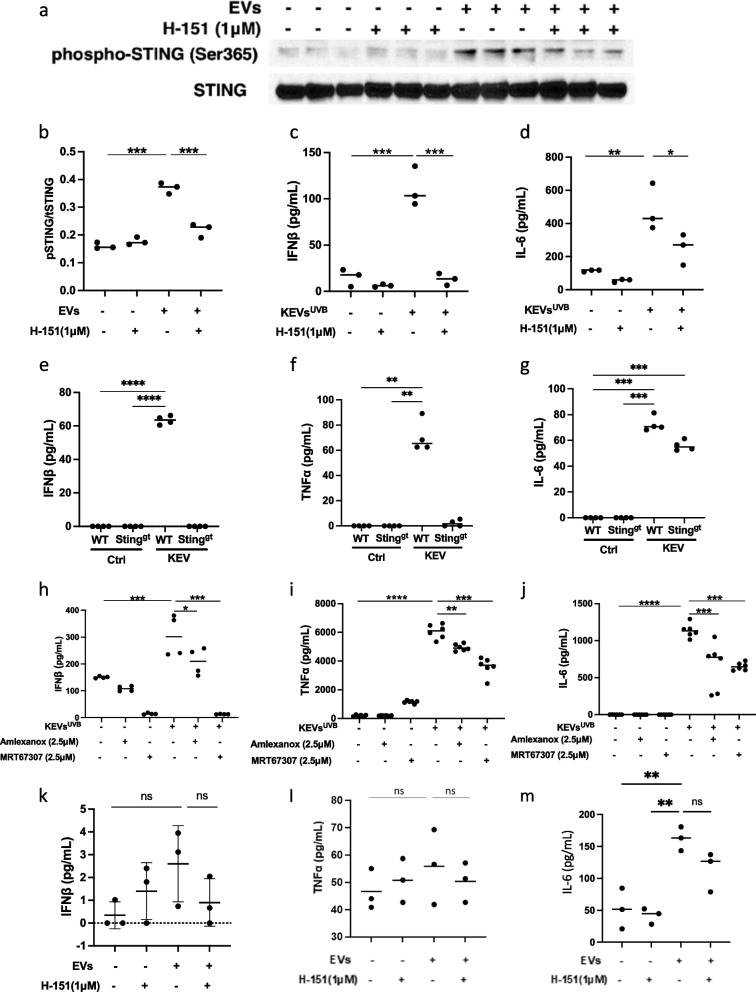
KEVs-UVB induced STING-dependent proinflammatory responses in macrophages and to a lesser extent in fibroblasts.UVB-induced KEVs stimulate STING phosphorylation (**a**, **b**), IFNß (**c**) and IL-6 (**d**) in RAW264.7 cells, while H-151inhibited those effects. UVB-induced KEVs stimulate WT murine macrophages to secrete more IFNß (**e**), TNFα (**f**), and IL-6 (**g**) than STING KO macrophages. TBK1 inhibitors suppressed UVB-induced KEVs-stimulated RAW264.7 macrophages secretion of IFNß (**h**), TNFα (**i**), and IL-6 (**j**). UVB-KEVs did not trigger more IFNβ or TNFα production in human primary dermal fibroblasts (**k**, **l**). UVB-KEVs did trigger increased production of IL-6 in fibroblasts, but the production was not attenuated by STING inhibition (**m**). Data represent mean±SD. *p<0.05, **p<0.01, ***p<0.001 between groups as indicated

UVB-induced KEVs stimulated STING phosphorylation in RAW264.7 cells. Pretreatment of RAW264.7 macrophages with 1μM of the STING antagonist H-151 significantly suppressed the ability of KEVs to induce STING phosphorylation (Fig. 3a,b). In addition, UVB-induced KEVs stimulated the macrophages to secrete proinflammatory cytokines, and H-151 inhibited KEVs-triggered cytokine release (Fig. 3c,d). Comparing the responses in macrophages isolated from WT and STING KO mice, UVB-induced KEVs triggered more proinflammatory cytokines release in WT macrophages than STING KO macrophages (Fig. 3e-g). TBK1 works downstream of STING, and the TBK1 inhibitors Amlexanox and MRT67307 suppressed KEVs-induced production of proinflammatory cytokines in macrophages (Fig. 3h-j).

### UVB irradiation released KEVs triggered less proinflammatory response in fibroblasts

In addition to macrophages, fibroblasts were also used to test KEVs-UVB induced proinflammatory responses. Unlike macrophages, KEVs-UVB failed to induce IFNβ or TNFα production in primary human fibroblasts. Interestingly, production of IL-6 was increased, and inhibition of the STING pathway with H-151 did not significantly attenuate IL-6 levels (Fig. 3k-m).

### UVB irradiation induced less dermal inflammation in STING KO mice than in WT mice

**Fig. 4:**
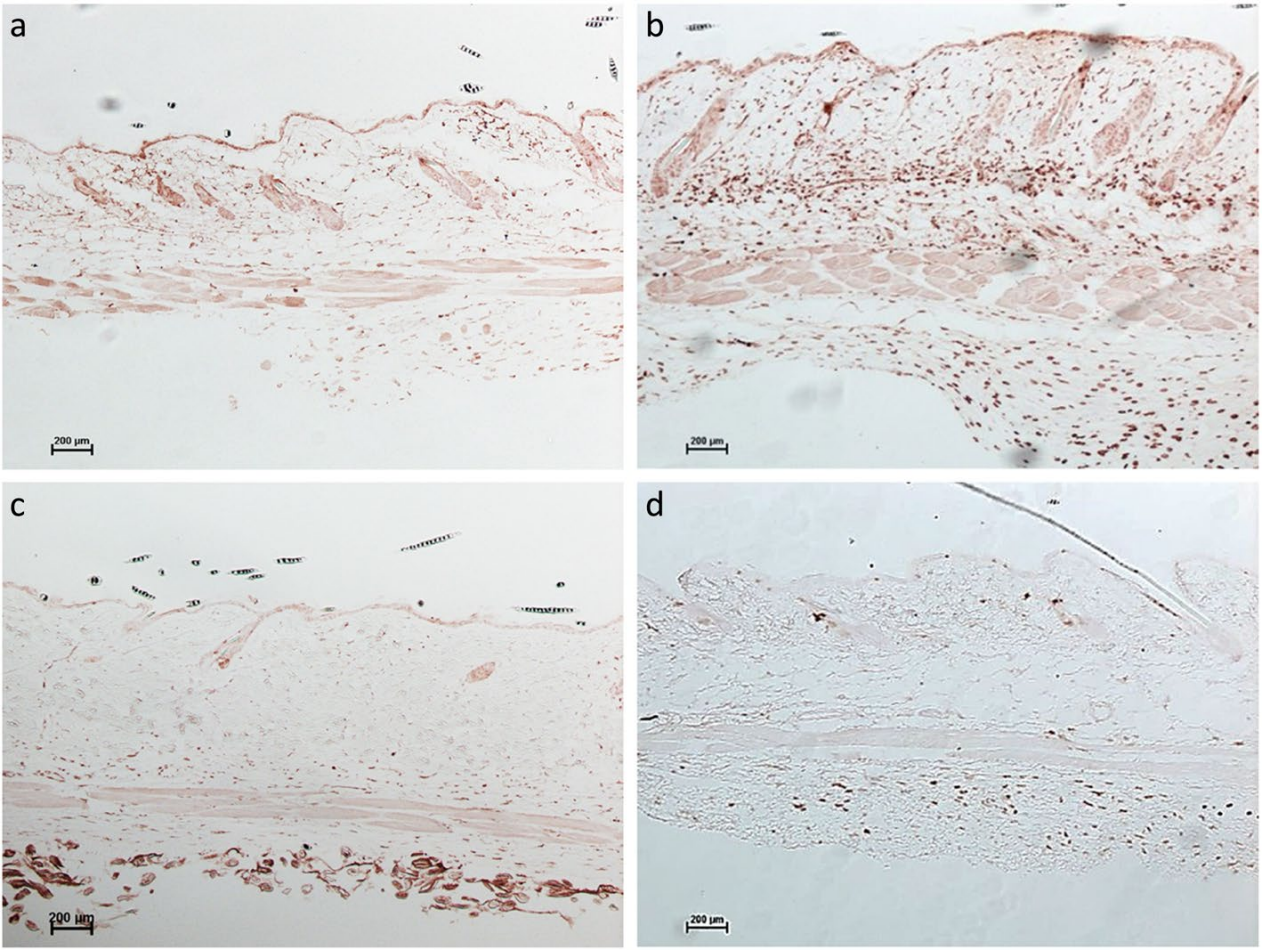
UVB irradiation induced less dermal inflammation in STING KO (Sting^gt/gt^) mice than in wild-type (WT) mice. CD45 staining shows that UVB irradiation of STING KO mice stimulated the recruitment of fewer immune cells into the dermis than in WT mice. **a** WT-CTRL (**b**) WT-UVB, **c **Sting gt/gt - CTRL (**d**) Sting gt/gt -UVB

To further test the role of STING in response to UVB in vivo, the dorsal skin of WT mice and STING KO mice were irradiated with zero (sham) or 150 mJ/cm^2^ of UVB daily for 5 days. Fewer numbers of inflammatory cells were recruited and accumulated in the dermis in UVB-irradiated STING KO mice (Fig. 4d) than in UVB-irradiated WT mice (Fig. 4b).

### Inhibition of STING signaling pathway suppressed UVB-induced KEVs triggered inflammasome activation in macrophages

**Fig. 5:**
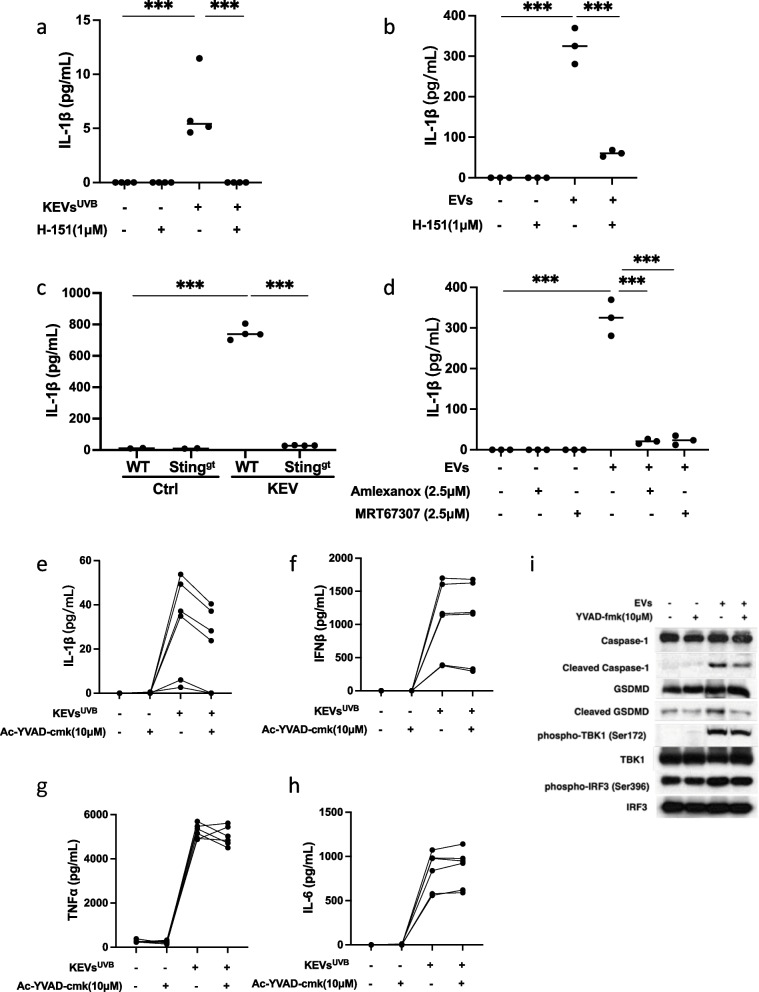
Suppression of STING and inflammasome pathways. Pretreatment of macrophages with the STING antagonist H-151 abolished KEVs-induced IL-1β release in primary mice macrophages (**a**) and RAW264.7 macrophages (**b**). KEVs-UVB induced production of interleukin-1 β is suppressed in STING KO macrophages (**c**). Pretreatment of macrophages with the TBK1 antagonists suppressed KEVs-UVB-induced IL-1β release (**d**). UVB-induced KEVs stimulated RAW264.7 cells to secrete large amounts of IL-1β, IFNß, TNFα, and IL-6. Pretreatment of the macrophages with 0μM (–) or 10μM (+) of the pyroptosis inhibitor Ac-YVAD-cmk suppressed KEVs-UVB-triggered IL-1β production (**e**) but had no effect on KEV-stimulated production of the other cytokines (**f**-**h**). Similar results obtained using VX765, a second inflammasome inhibitor, are shown in Supplementary Figure 1. Immunoblot shows inhibition of the inflammasome pathway affects apoptotic inflammasome markers but does not inhibit the STING pathway (**i**)

UVB-irradiated keratinocyte-derived EVs stimulated macrophages to produce the inflammasome-related cytokine interleukin-1β. Moreover, pretreatment of macrophages with 1μM of the STING antagonist H-151 abolished KEVs-induced IL-1β release in both primary mice macrophages (Fig. 5a) and RAW264.7 macrophages (Fig. 5b). Furthermore, KEVs-UVB induced more interleukin-1β release from WT macrophages than STING KO macrophages (Fig. 5c). TBK1 inhibitors also suppressed KEVs-UVB-induced interleukin-1 β release (Fig. 5d).

### Inhibition of inflammasome suppressed UVB-induced KEVs triggered inflammasome activation but had no effect on UVB-induced KEVs-triggered STING signaling pathway activation in macrophages

To explore the regulatory relation between the STING signaling pathway and the inflammasome pathway, two inflammasome inhibitors VX765 and Ac-YVAD-cmk were evaluated for their effect on KEVs UVB-induced proinflammatory responses in macrophages. As shown in Fig. 5e and Supplementary Figure 1, both VX765 and Ac-YVAD-cmk suppressed KEVs-UVB-triggered interleukin-1β production in macrophages, while these inflammasome inhibitors had no effects on KEVs-UVB-induced IFNβ, TNFα, and IL6 production (Fig. 5f-h and Supplementary Figure 1). Immunoblot results show that the two inflammasome inhibitors VX765 and Ac-YVAD-cmk could suppress KEVs-UVB increased caspase-1 and GSDMD cleavage but had no effects on phosphor-TBK1 and phosphor-IRF3 increase (Fig. 5i).

### Melatonin (M) suppressed UVB irradiation-induced KEVs release, apoptosis, skin inflammation, and KEVs-mediated proinflammatory responses

**Fig. 6:**
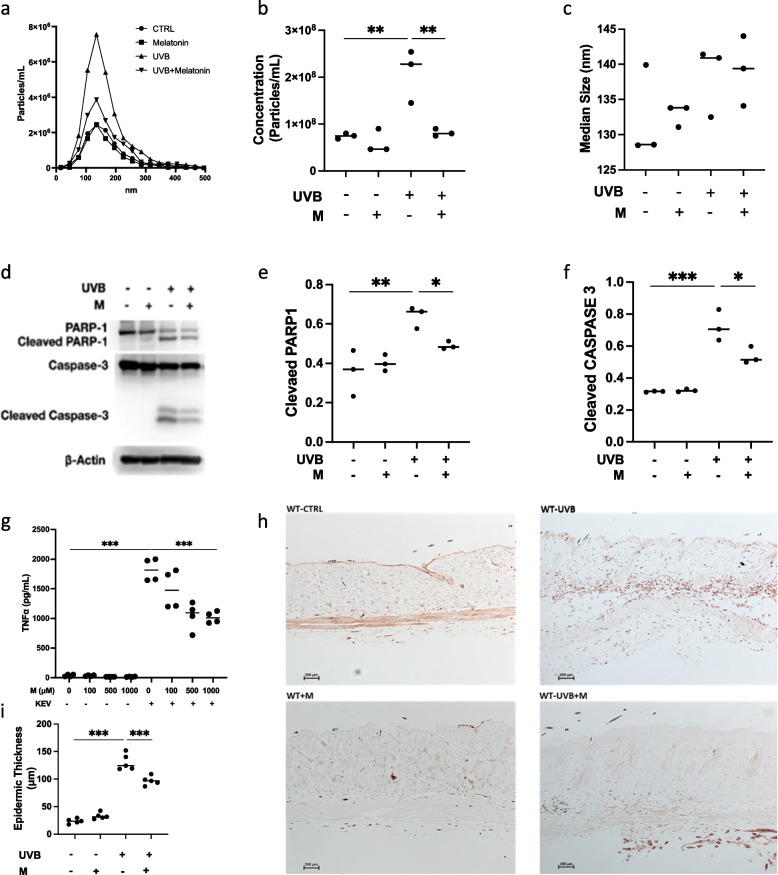
Melatonin suppressed UVB irradiation induced KEVs release, apoptosis, inflammation, and KEVs-mediated proinflammatory responses. HaCaT cells pretreated with melatonin produce a lower number of EVs compared with control keratinocytes (**a**, **b**). Particle mean size was not significantly different (**c**). Treatment of primary keratinocytes resulted in similar findings (Supplementary Figure 2). PARP-1 and Caspase-3 cleavage in primary keratinocytes is attenuated by melatonin (**d**-**f**). TNFα production by macrophages stimulated by UVB-induced KEVs is attenuated by pretreatment of macrophages with melatonin in a dose dependent manner (**g**). Skin biopsy of C57BL/6J mice treated with melatonin prior to UVB irradiation, stained for CD45, shows less inflammatory infiltrate (**h**) and thinner epidermis (**i**). Data represent mean ± SD. *<0.05, **p<0.01, ***p<0.001

Release of EVs by HaCaT or primary keratinocytes was suppressed by pretreatment with melatonin (Fig. 6a,b and Supplementary Figure 2) but did not affect their size (Fig. 6c). Melatonin pretreatment also impaired UVB irradiation-induced apoptosis as demonstrated by decreased PARP-1 and caspase-3 cleavage in primary keratinocytes (Fig. 6d-f). UVB-induced KEVs also strongly stimulated macrophage production of TNFα, that was attenuated by melatonin, while IFNβ or IL-6 were not attenuated (Fig. 6g and Supplementary Figure 3). In vivo, pretreatment of C57BL/6J mice with melatonin ameliorated UVB-induced skin inflammation as demonstrated by decreased dermal infiltrates (Fig. 6h). Finally, the epidermis of melatonin-treated mice was thinner (Fig. 6i).

## Discussion

In this study we show that UV-irradiated KEVs display a different profile compared to EVs derived from cells not exposed to UVB. UVB-irradiated keratinocytes secrete a higher number of EVs, their cargo is richer in proteins and in DNA, and they have an immunostimulatory function. Immune activation is mediated by the STING and inflammasome pathways, while the latter functions downstream of the former. Pyroptosis was not affected by KEVs. In addition, melatonin can reverse inflammation and apoptosis induced by UVB-irradiated KEVs.

We and others have pointed out that composition, and hence function, of EVs vary considerably, depending on the circumstances of their generation and their cells of origin [28]. EVs may both induce or suppress inflammation. EVs formed under normal or only mildly stressful conditions can exert therapeutic roles. For example, fibronectin-containing EVs protected melanocytes against UVB-induced cytotoxicity [7]. EVs from adipose-derived stem cells ameliorated UVB-induced skin photoaging by attenuating inflammation and production of reactive oxygen species [46]. Mesenchymal stem cell-derived EVs protected mice skin against oxidative stress induced by UV-irradiation via the nuclear factor-erythroid 2-related factor-2 (Nrf2) signaling pathway [43], the same pathway that was found to mediate attenuation of the inflammasome by melatonin in glial cells. As for keratinocytes, the major component of the epidermis, less is known. Recent studies show that KEVs induced by low-dose, but not high-dose, UVB protected Schwann cells against toxicity from high concentrations of glucose [42]. KEVs that contained platelet activating factor (PAF) mediated UVB-induced systemic immunosuppression, noted by a decrease in the level of inflammatory cytokines such as TNFα, IL-1 and IL-6 in KEVs [26]. On the other hand, our results presented herein show that KEVs are capable of inducing increased production of these cytokines by macrophages, implying KEVs may have a proinflammatory function. Our published data show that UVB-irradiation recruits neutrophils to the dermal-epidermal junction and triggers the formation of neutrophil extracellular traps (NETs) in vitro and in vivo [25]. Given that PAF induced the formation of NETs from both human and murine neutrophils, this effect might be mediated by PAF released by UVB-irradiated KCs [25]. Additional evidence for a proinflammatory effect of KEVs is reported in several studies. UVB-irradiation of keratinocytes resulted in a dose-dependent increase in EV release and production of the proinflammatory cytokine IL-8, both attenuated by blockage of the same enzymatic path, implying vesicle release and IL-8 production are related [6]. The EV-mediated crosstalk within the epidermis is mediated not only by cytokines or other protein content, but also by nucleic acids. A recent study reported the presence of cyclobutane pyrimidine dimers, a DNA crosslink product formed due to photodamage, in EVs derived from UVB-irradiated keratinocytes, and its uptake by bystander keratinocytes [9]. Stimulation of fibroblasts by UVB-KEV did not lead to an increase in TNFα or INFβ levels but did lead to increased production of IL-6. Interestingly, an increase in IL-6 levels had been demonstrated in fibroblasts stimulated with non-irradiated KEVs [19] and the effect of UVR remains to be studied.

Several studies, including our own [24], have reported that certain EVs activate the STING signaling pathway. EVs released by herpes simplex virus (HSV) 1-infected cells blocked viral replication in recipient cells in a STING-dependent manner [12]. DNA-containing EVs from activated T cells primed dendritic cells through antigen-driven contacts [40]. We found that EVs from the plasma of patients with dermatomyositis, a photosensitive autoimmune disease, induced STING-mediated proinflammatory responses in target peripheral blood mononuclear cells (PBMCs) [24]. A different STING antagonist that acts by modulating the interaction of STING with stromal interaction molecule 1 (STIM1), thereby blocking its trafficking from ER to Golgi, inhibited the progression of lupus, another photosensitive autoimmune disease, in human patients. This antagonist suppressed the ability of EVs from the plasma of these patients to trigger proinflammatory responses in healthy PBMCs [33], similar to our findings in dermatomyositis.

Published work indicates that UVB irradiation of KCs induces DNA damage and presumably leakage into the cytosol, leading to activation of the cGAS-STING pathway and then STING-dependent apoptosis of the KCs [23, 37]. Apoptotic blebbing of UVB-irradiated KCs may contribute to UVB-induced KEVs. Moreover, it is possible that these apoptotic blebs will contain active components of the cGAS-STING pathway that could be delivered to target immune cells in the dermis. How UVB-induced KEVs mediate activation of the cGAS-STING pathway in target cells remains to be studied.

Several studies have highlighted the crosstalk between STING signaling pathway and inflammasomes in the innate immune system. A recent study found that STING promoted NLR family pyrin domain containing 3 (NLRP3) localization into the ER and facilitated NLRP3 deubiquitination to activate the inflammasome after HSV-1 infection [44]. Using different models, other groups reported that a STING-dependent cell death program upstream of NLRP3 activated the inflammasome in human myeloid cells [17]. Targeting the cGAS-STING lysosomal cell death (LCD)-NLRP3 pathway might ameliorate pathology in inflammatory conditions associated with sensing of cytosolic DNA [17]. The cytosolic DNA-STING-NLRP3 axis is also involved in murine acute lung injury induced by lipopolysaccharide [32], and the human-specific STING agonist G10 could activate type I interferon and the NLRP3 inflammasome [30]. In the other direction, activation of the absent in melanoma 2 (AIM2) inflammasome in murine macrophages and dendritic cells in vitro led to reduced activation of the STING pathway, in part through promoting caspase-1-dependent cell death [11]. Our data suggest that UVB-induced EVs have a role in activation of the inflammasome that is downstream of the STING pathway.

Melatonin and its metabolites exhibit antiproliferative and pro-differentiation properties in human epidermal KCs [20]. These effects have been described mainly in intact cultured skin or in immortalized human keratinocytes (HaCaT cells) [45]. In a model of accelerated aging and neurodegeneration, melatonin inhibited the ability of mtDNA in the cytosol to activate the cGAS-STING-IRF3 inflammatory pathway [21]. Melatonin has protective activity on lupus nephritis that is associated with its effect on enhancing antioxidant signaling and decreasing renal NLRP3 inflammasome activation [8]. Similarly, melatonin attenuated lipopolysaccharide induced NLRP3 inflammasome activation in microglia via the Nrf2 signaling pathway [2]. Our data, in agreement with this previous literature, supports a protective role of melatonin in the skin, specifically ameliorating inflammation and apoptosis. When treated with melatonin, UVB-irradiated keratinocytes produced a significantly lower number of EVs, providing one possible mechanistic explanation to the protective effect of melatonin.

UVR is a known inducer of replicative senescence in the skin [4]. A recent study demonstrated that EVs secreted from senescent dermal fibroblasts had a proinflammatory effect on keratinocytes [10]. The role of EVs in UV-induced senescence in the epidermis remains to be studied.

Further studies exploring EVs, especially KEVs induced by damaging doses of UVB, and their impact on other cells will provide insight into photodamage and may help develop targeted therapeutic approaches.

## Materials and methods

### Cell culture

Human keratinocyte cell line HaCaT, kindly provided by Dr. John T. Seykora, and RAW 264.7 macrophage, purchased from ATCC, were grown in 75 cm^2^ cell culture flasks and maintained in high-glucose DMEM, supplemented with 10% fetal bovine serum (FBS), L-glutamine, and antibiotic/antimycotic solution containing 100 U/mL penicillin, 100μL/mL streptomycin, and amphotericin B at 37℃ in a humified incubator with 5% CO_2_. Neonatal Human Epidermal Keratinocytes (NHEK) were grown in 10cm petri dish and maintained in Kc 50/50, that is an equal mixture of 2 commercially available media: K-SFM combo (Life Technology 17005042) and Media 154 (Life Technology M154500, 200uM CaCl_2_) plus their supplements including 100 U/ml Penn, 100 µg/ml Strep and .25 µg/mL Fungizone. Mice peritoneal macrophages isolation was conducted with 3% thioglycolate broth (TGB) medium induction and then isolated as previously described [48]. Cells were maintained in RPMI-1640 medium with 10% heat-inactivated FBS, 2mM L-glutamine, 100 U/mL of penicillin, and 100 μg/mL of streptomycin. Primary human keratinocytes and primary human fibroblast were provided by Penn Skin Biology and Diseases Resource-based Center (SBDRC). The following plates were used: Multi well Culture Plate, PS, 6 wells, External Dimensions wxlxh85.40x127. 60x20. 20mm, Well Dimensions dxh 35.00x17. 50mm, Culture Area 9.60cm^2^, Working Volume 3.00 ml, TC Treated, Sterile to SAL 10-6,1/Bag, 50/Case.

### Primary human macrophage differentiation

PBMCs isolated from fresh blood of healthy donor were prepared from buffy coats using Ficoll-Paque (GE 45-001-749). Monocytes were enriched from PBMCs by Monocyte Attachment Medium (PromoCell 50306290). The monocytes were expanded in culture for 7 days in the presence of 100 ng/mL human GM-CSF (Peprotech 300-03).

### Extracellular vesicles irradiation, isolation and identification

Extracellular vesicles derived from keratinocytes were isolated and purified by the classical differential centrifugation and ultracentrifugation method according to the MISEV2018 guideline. Keratinocytes (2.5*10^6^ cell/mL of HaCaTs and 0.5*10^6^ cells/mL of NHEKs) were grown to 90% confluence, washed with PBS and irradiated with two FS40T12 UVB bulbs (Light of America, Walnut CA), output of 0.22 mW/cm^2^ with peak irradiation at 313 nm equipped with a cellulose triacetate filter to remove wavelengths below 290 nm at 75 mJ/cm^2^ for HaCaT cells and 30 mJ/cm^2^ for NHEKs. The source medium was then returned to the cells and cultured for 18 hours. The medium was collected and centrifuged at 500xg for 10 min, followed by 2000xg for 20 min to remove cells, cellular debris, and apoptotic bodies. The supernatant was then filtered through a 0.22μm sterile filter. The filtered supernatant was then ultracentrifuged at 100000xg for 120 min at 4℃ in an ultracentrifuge (Beckman Coulter) to collect the pellets. After removing the supernatant, the extracellular vesicles were washed with sterile PBS and then ultracentrifuged at 100000xg for 60 min. The pellets were finally resuspended, aliquoted, and used immediately for the experiments or stored at -80℃. Cell viability was determined using trypan blue and a cell counter (Clay Adams, USA). The concentration and size distribution of the EVs were analyzed on ZetaView ParticleMetrix, Germany). The protein concentration in the extracellular vesicles was measured by using a Pierce Bicinchoninic Acid (BCA) Protein Assay Kit. DNA captured by extracellular vesicles was purified by using PureLink Genomic DNA Mini Kit (Invitrogen), and measured by using Nanodrop, according to the manufacturer’s instructions. Western blot analysis was used to determine extracellular vesicles surface marker CD81, cytosolic marker TSG101 and Calnexin as a negative marker.

### Immunoblot analysis

Radioimmunoprecipitation assay (RIPA) lysis buffer was used to lyse cells. After measured by BCA Protein Assay Kit, the equivalent amounts of protein were separated by SDS-PAGE, and then transferred to PVDF membranes. The membranes were blocked with 5% BSA in TBST buffer, and then incubated with primary antibodies at 4℃ overnight. The membranes were then washed and incubated with secondary antibody for 60 min, followed by addition of West Femto Maximum Sensitivity Substrate (Thermo Scientific), and then detected by LiCor. ImageJ was used to semi-quantify the density of the protein bands.

### Enzyme-linked immunosorbent assay (ELISA)

ELISA was performed to measure proinflammatory cytokines IFNβ, TNFα, and IL-6 levels by Huamn DuoSet ELISA kit (R&D systems, Minneapolis, MN) following manfacturer’s instructions. Briefly, peritoneal macrophages eluted from C57BL/6J (Stock No.:000664) and C57BL/6J-Sting1gt/J (Stock No.:017537) mice were stimulated with extracellular vesicles for 15 h, and then the supernatant was collected for ELISA.

### Animal experiments

C57BL/6J (Stock No.: 000664) and C57BL/6J-Sting1gt/J (Stock No.: 017537) mice purchased from the Jackson laboratory were housed in a pathogen-free environment and given food and water ad libitum. All mice participating in this experiment were female. All the animal experiments were approved by the Institutional Animal Care and Use Committee of Philadelphia VA Medical Center and conducted according to the NIH Guide for the Care and Use of Laboratory Animals. For the experiments with UVB exposure, the dorsal skin of mice was exposed to UVB with two FS40T12-UVB bulbs (Light Sources) according to our published protocol. STING KO mice and WT mice in 8 weeks of age were randomly assigned to pretreat in the presence/absence of 20 mg/kg body weight of melatonin administered via intraperitoneal route 3 hours prior to UVB exposure, then receive UVB exposure with or without 150 mJ/cm2 for five consecutive days under anesthetization by peritoneal injected Ketamine/Xylazine. Animals were sacrificed 24 h after the last exposure. The whole dorsal skin samples were collected and tissue sample sectioning was performed by the Penn SBDRC.

### Statistical analysis

Prism 8 (GraphPad Software) was used to perform statistical analysis. Normal distributed data were shown as mean ± standard deviations (SD). Comparisons between two groups were conducted with the Student t test and comparisons among three or more groups were performed by using ANOVA, followed by Student-Newman-Keuls test. Statistical significance was considered at a level of *P*-value < 0.05.

Reagents and antibodies are listed in the supplementary material.

## Supplementary Information

Supplementary Material 1.

Supplementary Material 2.

## Data Availability

No datasets were generated or analysed during the current study.

## Abbreviations

DAMPs: Damage-associated molecular patterns
EVs: Extracellular vesicles
KCs: Keratinocytes
KEV: KC-derived vesicles
PBMCs: Peripheral blood mononuclear cells
UVB: Ultraviolet B
UVR: Ultraviolet radiation
