# Supplementary material for: Keratinocyte derived extracellular vesicles mediated crosstalk between epidermis and dermis in UVB-induced skin inflammation
Source: Cell Commun Signal. 2024 Sep 30;22:461. doi: 10.1186/s12964-024-01839-9 (PMC11441254; doi:10.1186/s12964-024-01839-9)
Supplement: Supplementary file 1 — Supplementary Material 1. [file 12964_2024_1839_MOESM1_ESM.docx]

**
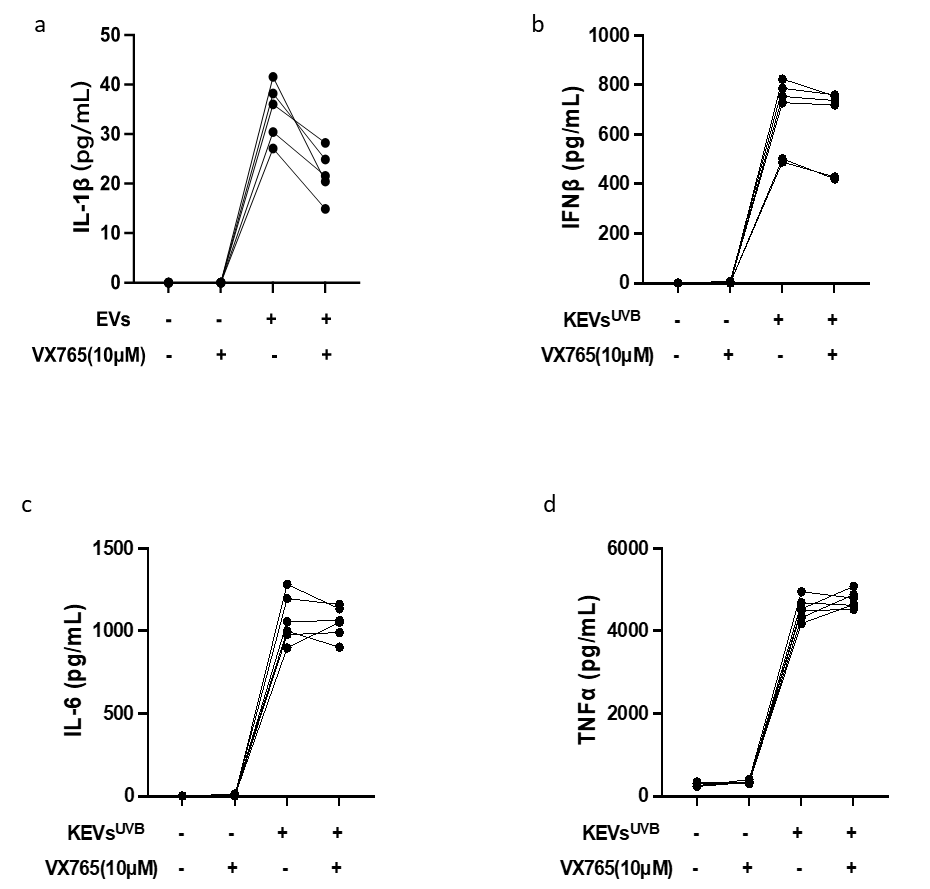
**

**Supplementary Figure 1. Suppression of the inflammasome pathways.** UVB-induced KEVs stimulated RAW264.7 cells to secrete large amounts of IL-1β, IFNß, IL-6, and TNFα. Pretreatment of the macrophages with 0μM (–) or 10μM (+) of the pyroptosis inhibitor VX765 suppressed KEVs-UVB-triggered IL-1β production (a) but had no effect on KEV-stimulated production of IFNß, IL-6, and TNFα and b-d).

**
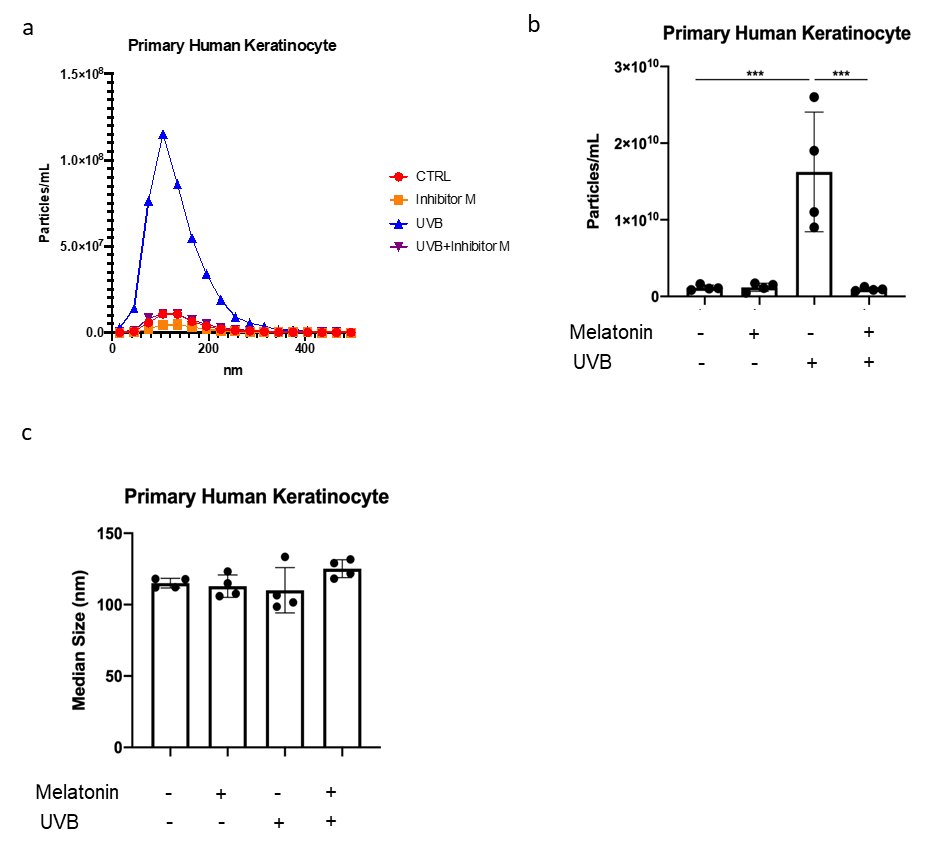
**

**Supplementary Figure 2. Suppression of UVB irradiation-induced KEVs release by melatonin.** Primary keratinocytes pretreated with melatonin produce a different size distribution (a) and a lower number (b) of EVs compared with control keratinocytes (a,b). Particle mean size was not of significant difference (c)

**
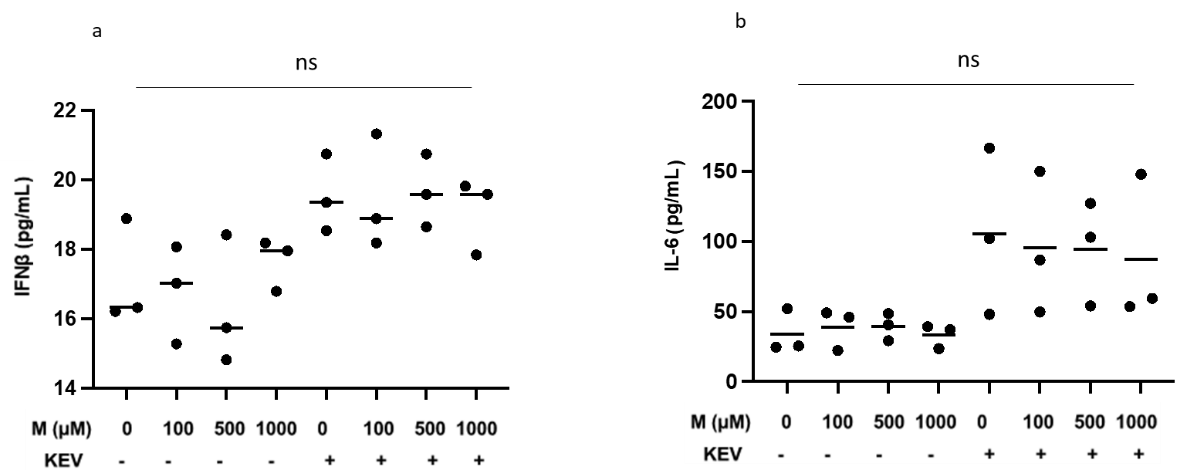
**

**Supplementary Figure 3. Melatonin had no effect on UVB-induced KEVs production of IFNβ or IL-6.** Production of the IFNβ (a) or IL-6 (b) by macrophages stimulated by UVB-induced KEVs is not attenuated by pretreatment of macrophages with melatonin.

**Supplementary text: Reagents and antibodies**

STING antagonist H-151, TBK1 inhibitors Amlexanox and MRT67307, inflammasome inhibitors VX765 and Ac-YVAD-cmk, and Melatonin were purchased from Cayman Chemical Company (Ann Arbor, MI). Phospho-STING (Ser365) (D8F4W) rabbit antibody, STING (D2P2F) rabbit antibody, phosphor-TBK1/NAK (Ser172) (D52C2) rabbit antibody, TBK1/NAK (D1B4) rabbit antibody, phospho-IRF-3 (Ser396) (D601M) rabbit antibody, IRF-3 (D83B9) rabbit antibody, and β-Actin (8H10D10) mouse antibody were obtained from Cell Signaling Technology Company (Danvers, MA). Rabbit anti-tumor TSG101 (T5701) was obtained from Sigma-Aldrich (St. Louis, MO), mouse anti-calnexin (clone E-10, catalogue number SC-46669) and mouse anti-CD81 (clone B-11, catalogue number sc-166029) were purchased from Santa Cruz (Dallas, TX). HRP conjugated goat anti-rabbit or mouse secondary antibody were obtained from Jackson ImmunoResearch Laboratories (West Grove, PA). Alexa Fluor 568 goat anti-mouse IgG and Alexa Fluor 568 goat anti-rabbit IgG secondary antibodies were obtained from Thermo Fisher (Eugene, OR). Antibody concentration: The primary antibody is 1:1000 dilution, and the secondary antibody is 1:10000.
