# Supplementary material for: Keratinocyte derived extracellular vesicles mediated crosstalk between epidermis and dermis in UVB-induced skin inflammation
Source: Cell Commun Signal. 2024 Sep 30;22:461. doi: 10.1186/s12964-024-01839-9 (PMC11441254; doi:10.1186/s12964-024-01839-9)
Supplement: Supplementary file 2 — Supplementary Material 2. [file 12964_2024_1839_MOESM2_ESM.pptx]

## Slide 1
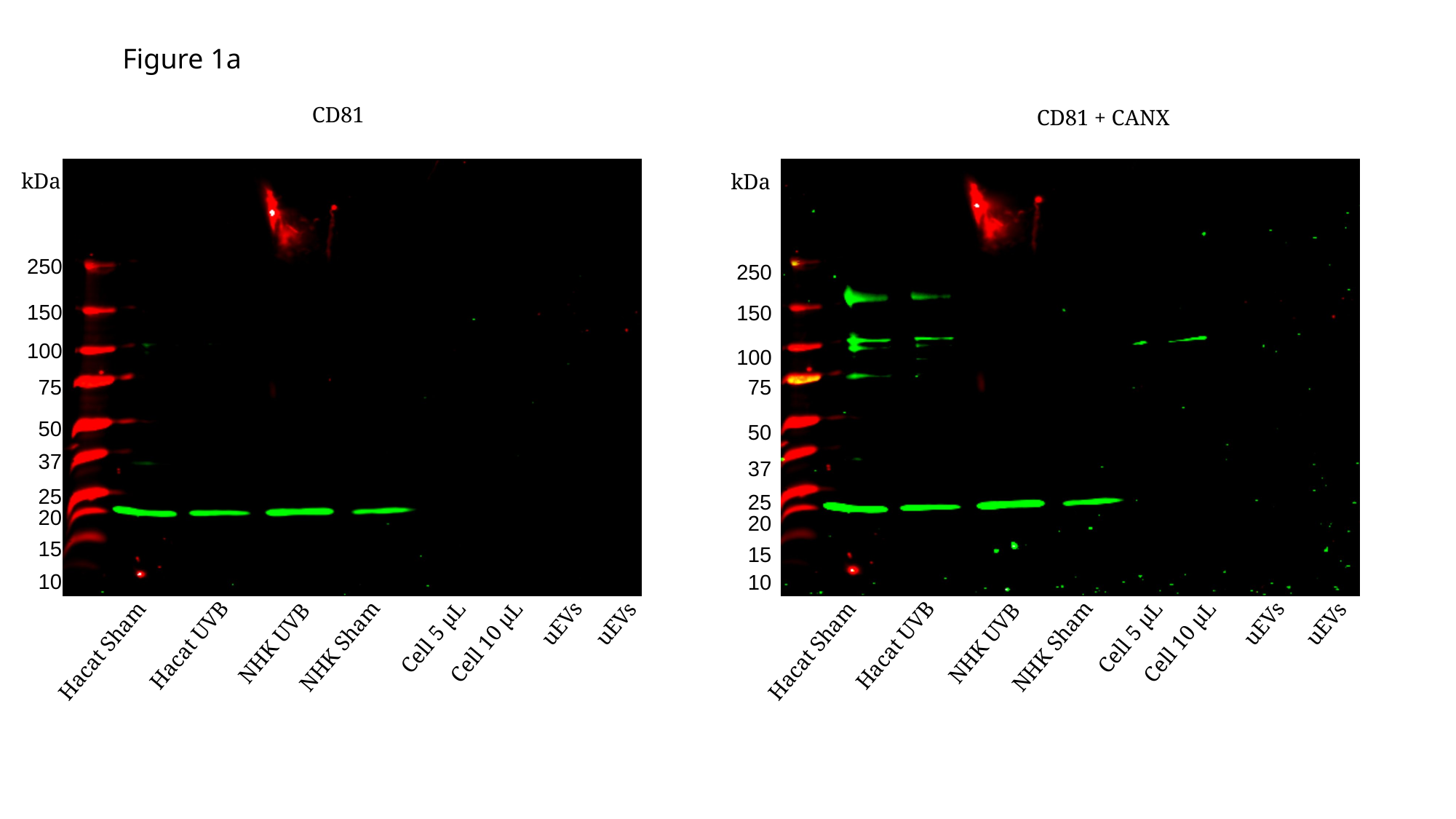

Figure 1a
CD81
CD81 + CANX
kDa
kDa
250
250
150
150
100
100
75
75
50
50
37
37
25
25
20
20
15
15
10
10
uEVs
uEVs
uEVs
uEVs
Cell 5 µL
Cell 5 µL
NHK UVB
NHK UVB
Hacat UVB
Hacat UVB
NHK Sham
NHK Sham
Cell 10 µL
Cell 10 µL
Hacat Sham
Hacat Sham

## Slide 2
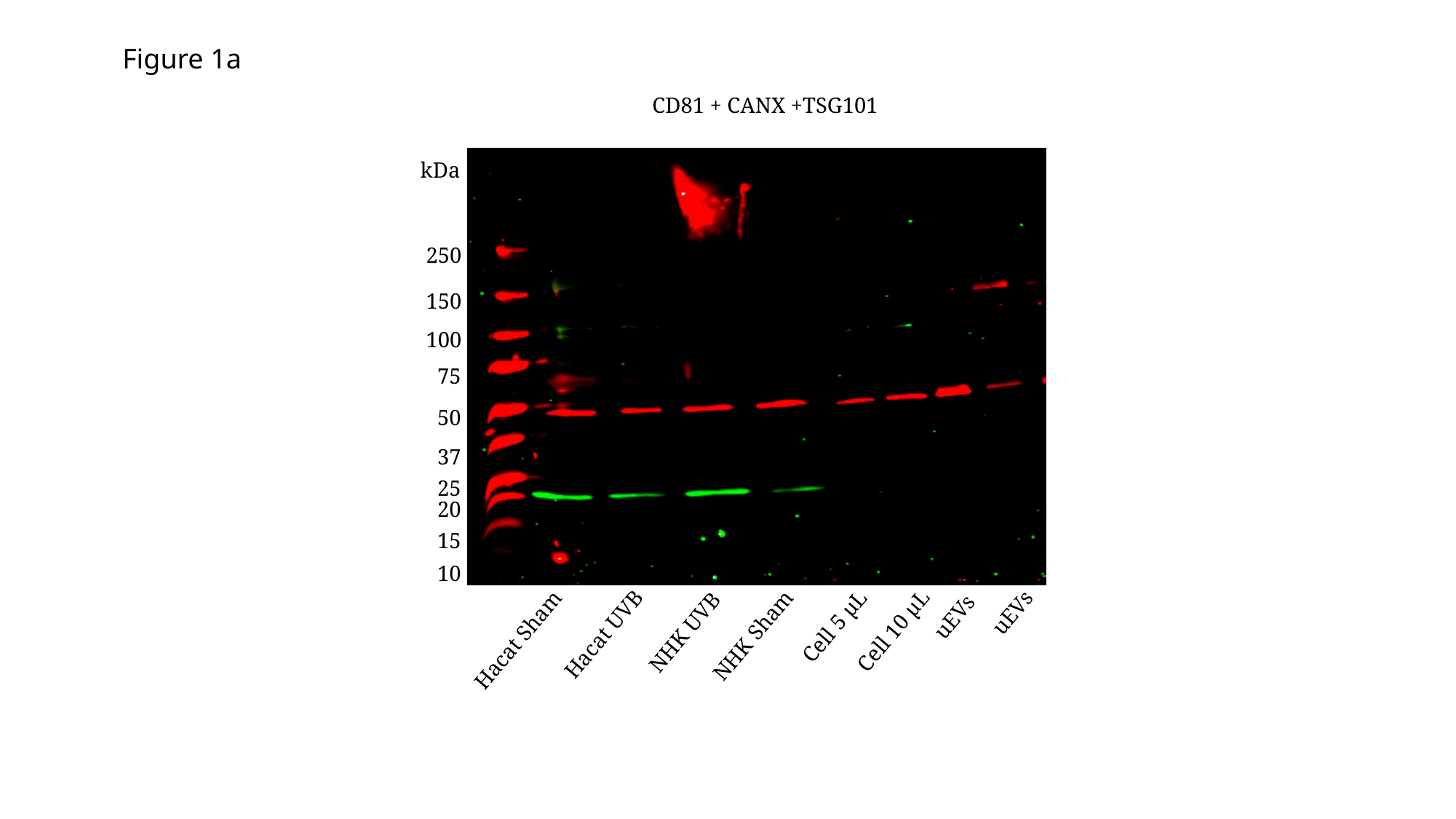

Figure 1a
CD81 + CANX +TSG101
kDa
250
150
100
75
50
37
25
20
15
10
uEVs
uEVs
Cell 5 µL
NHK UVB
Hacat UVB
NHK Sham
Cell 10 µL
Hacat Sham

## Slide 3
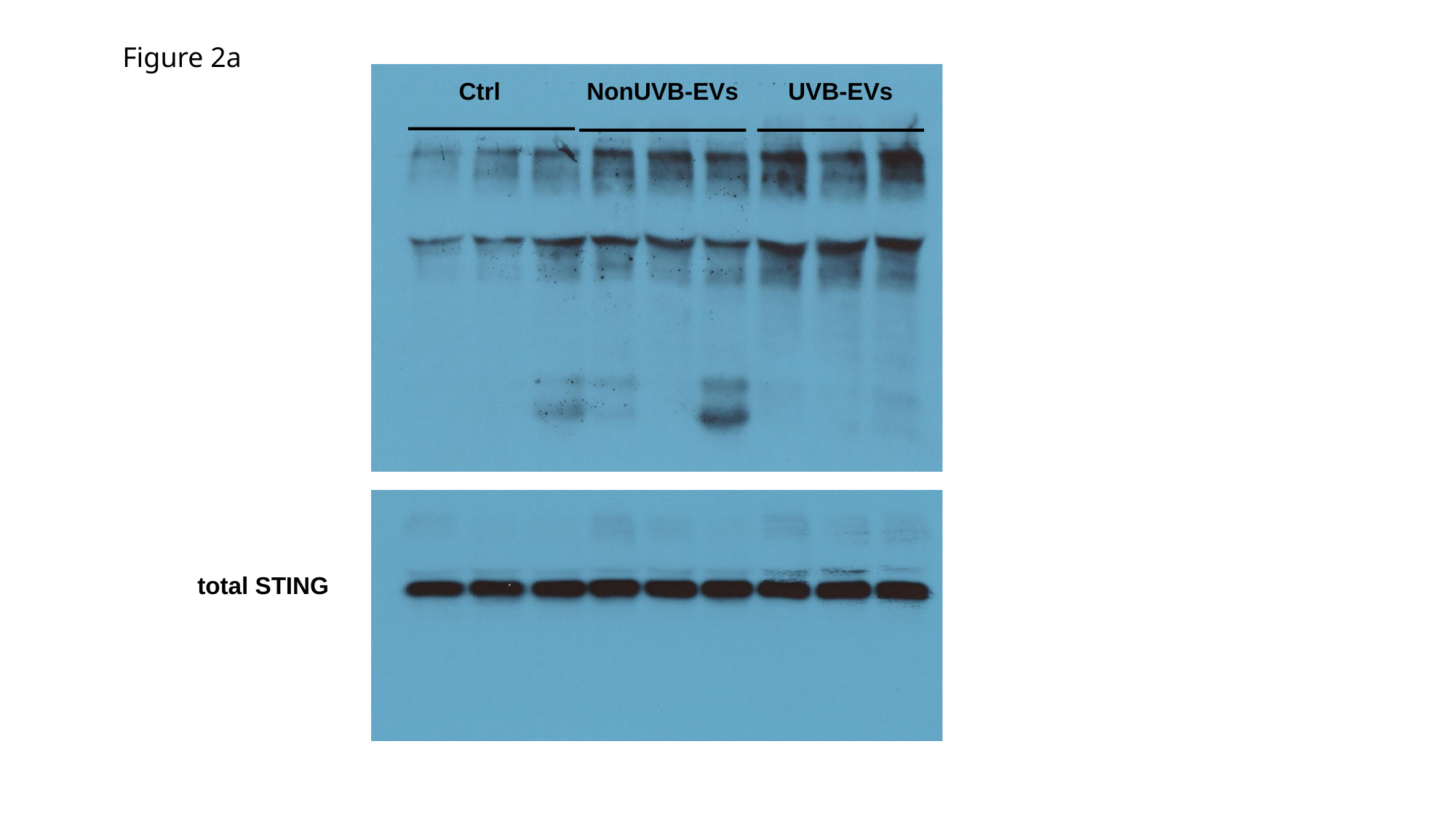

Figure 2a
Ctrl
NonUVB-EVs
UVB-EVs
total STING

## Slide 4
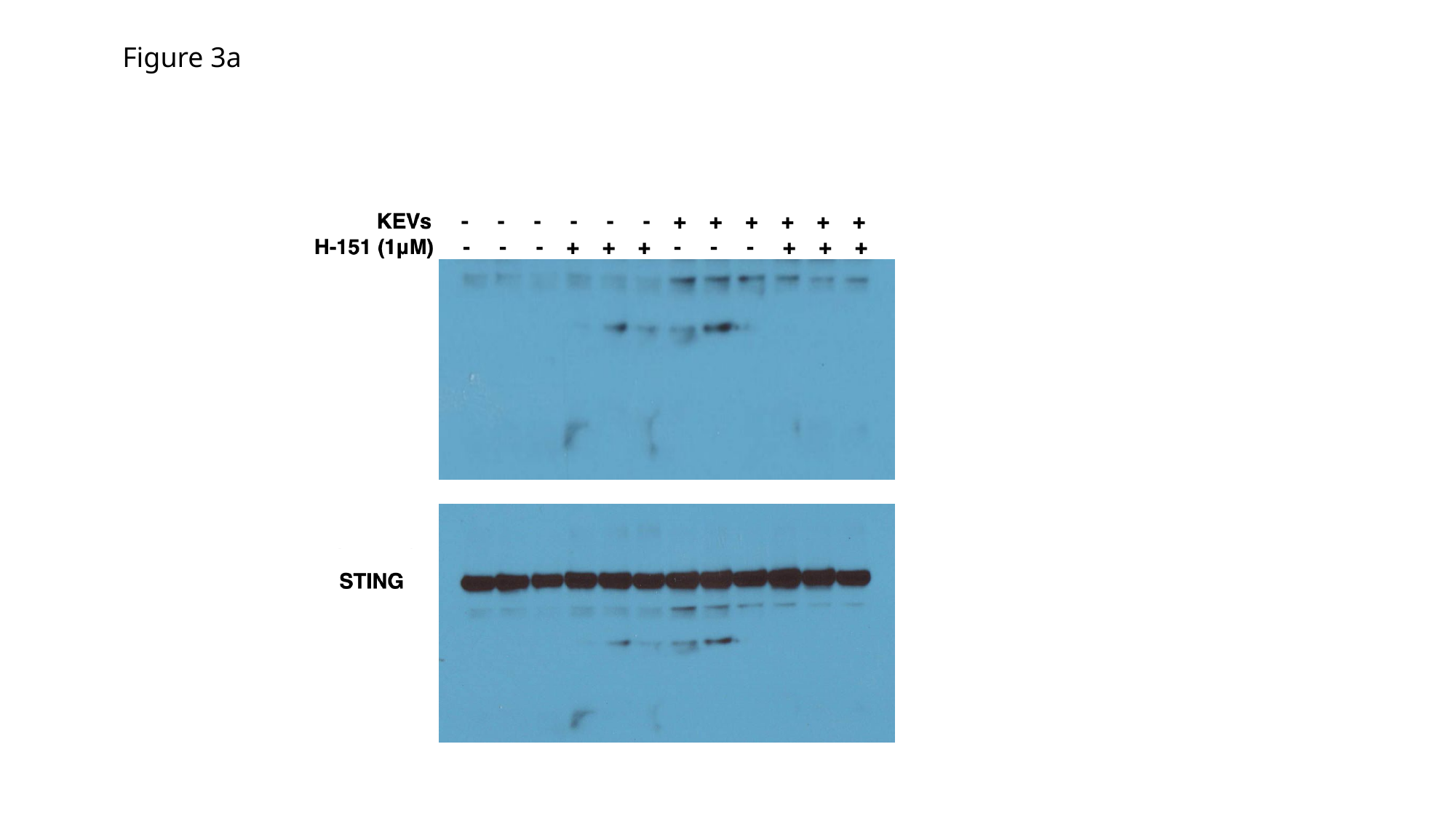

Figure 3a

## Slide 5
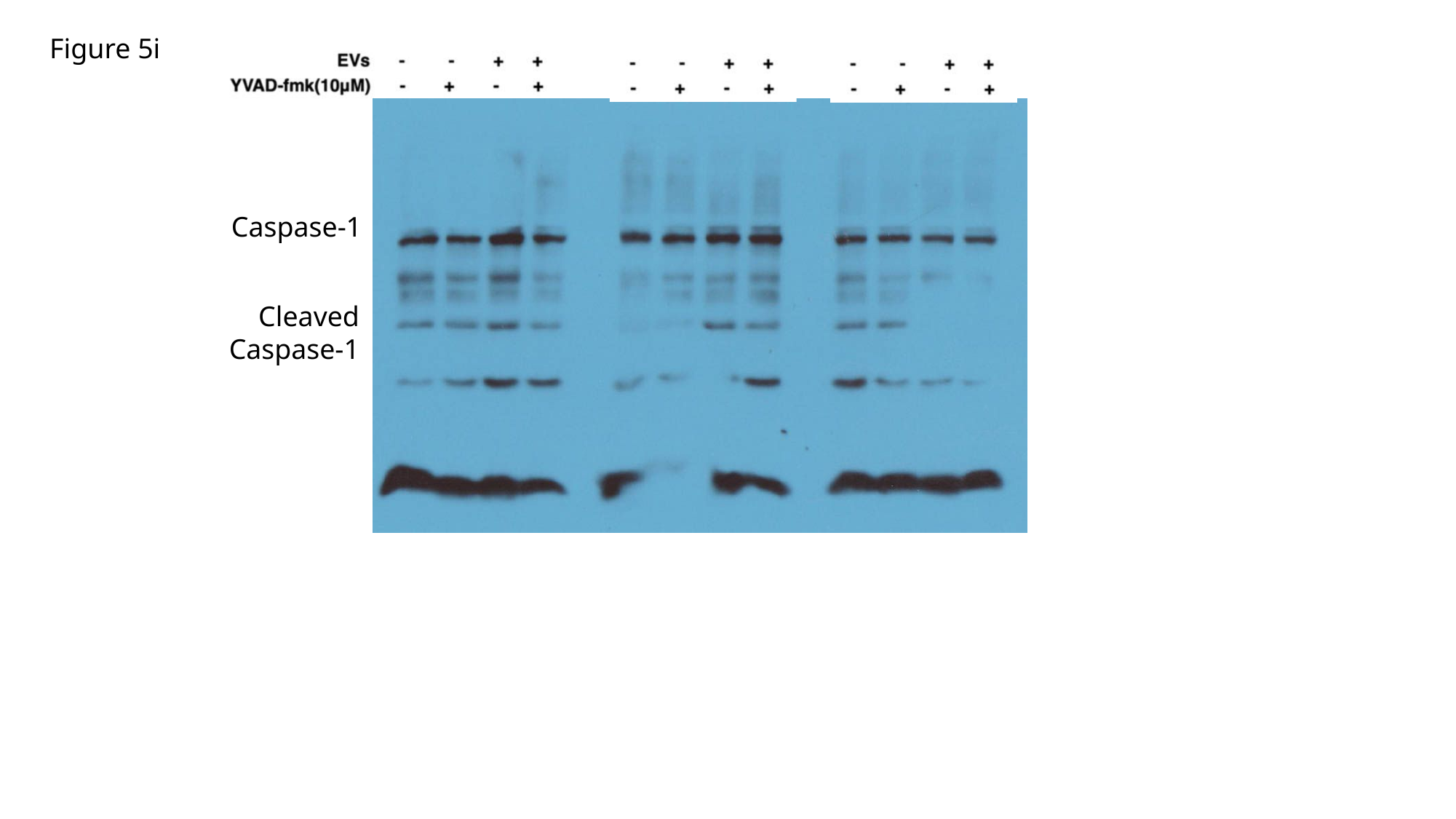

Figure 5i
Caspase-1
Cleaved Caspase-1

## Slide 6
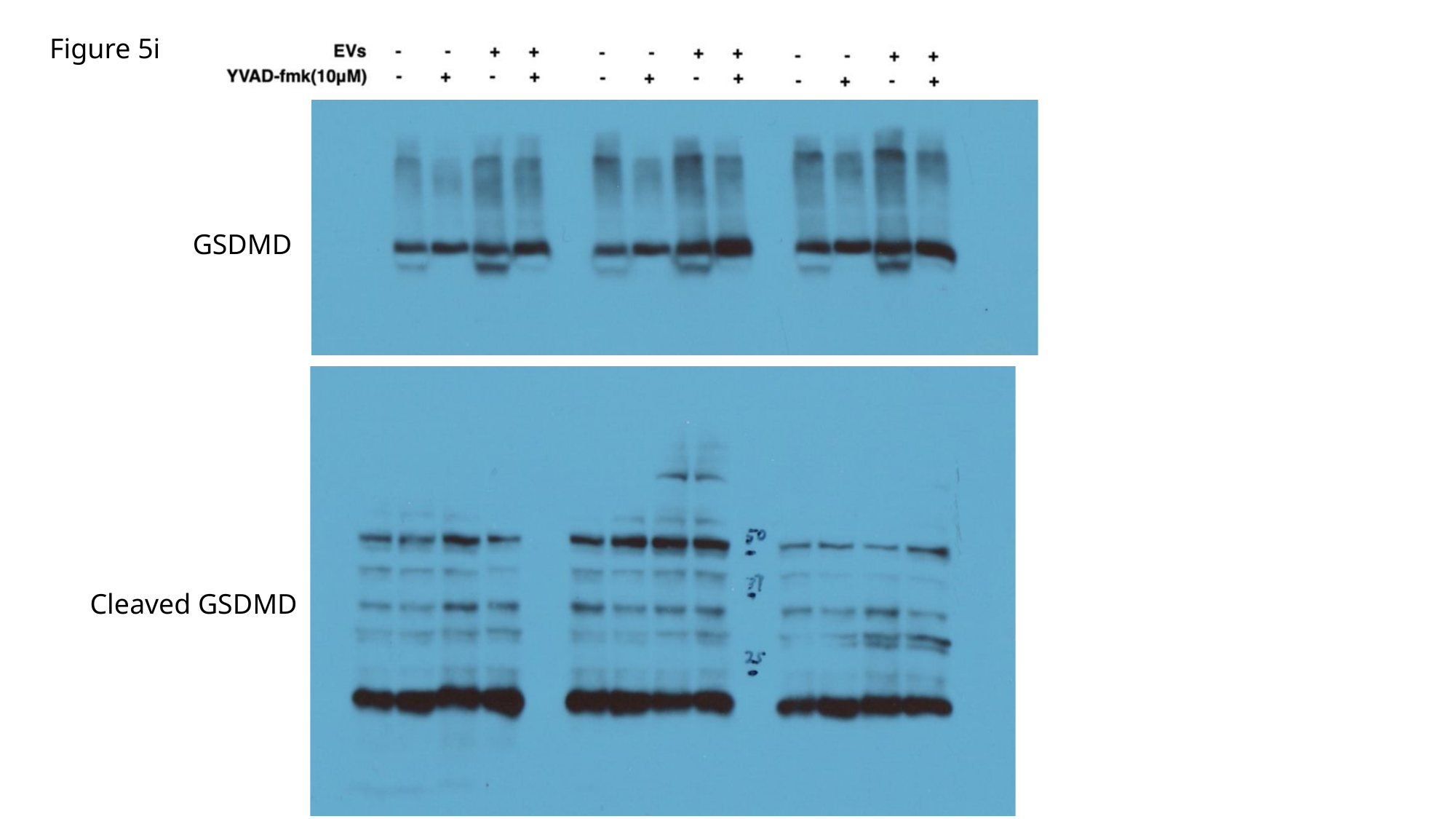

Figure 5i
GSDMD
Cleaved GSDMD

## Slide 7
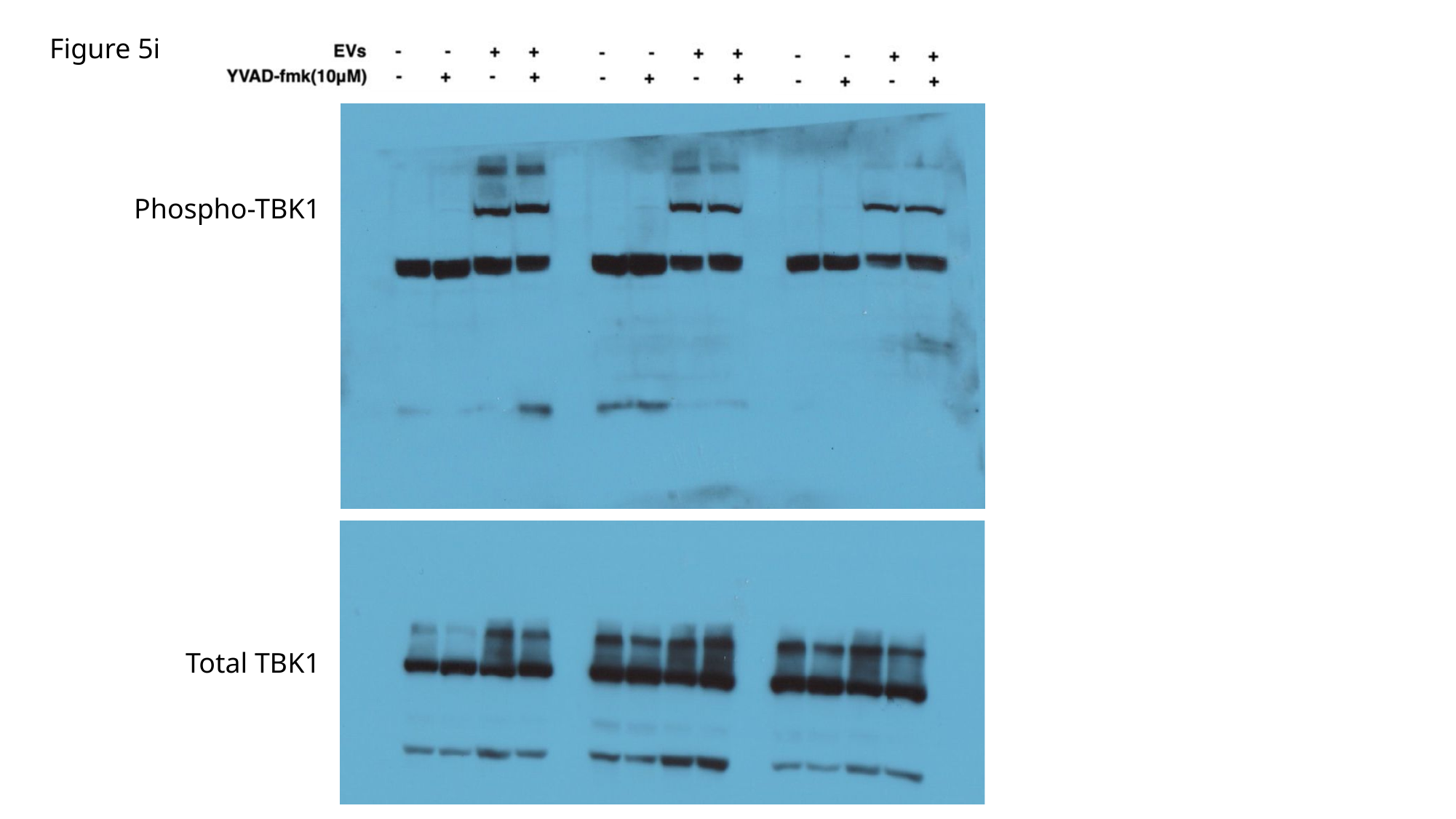

Figure 5i
Phospho-TBK1
Total TBK1

## Slide 8
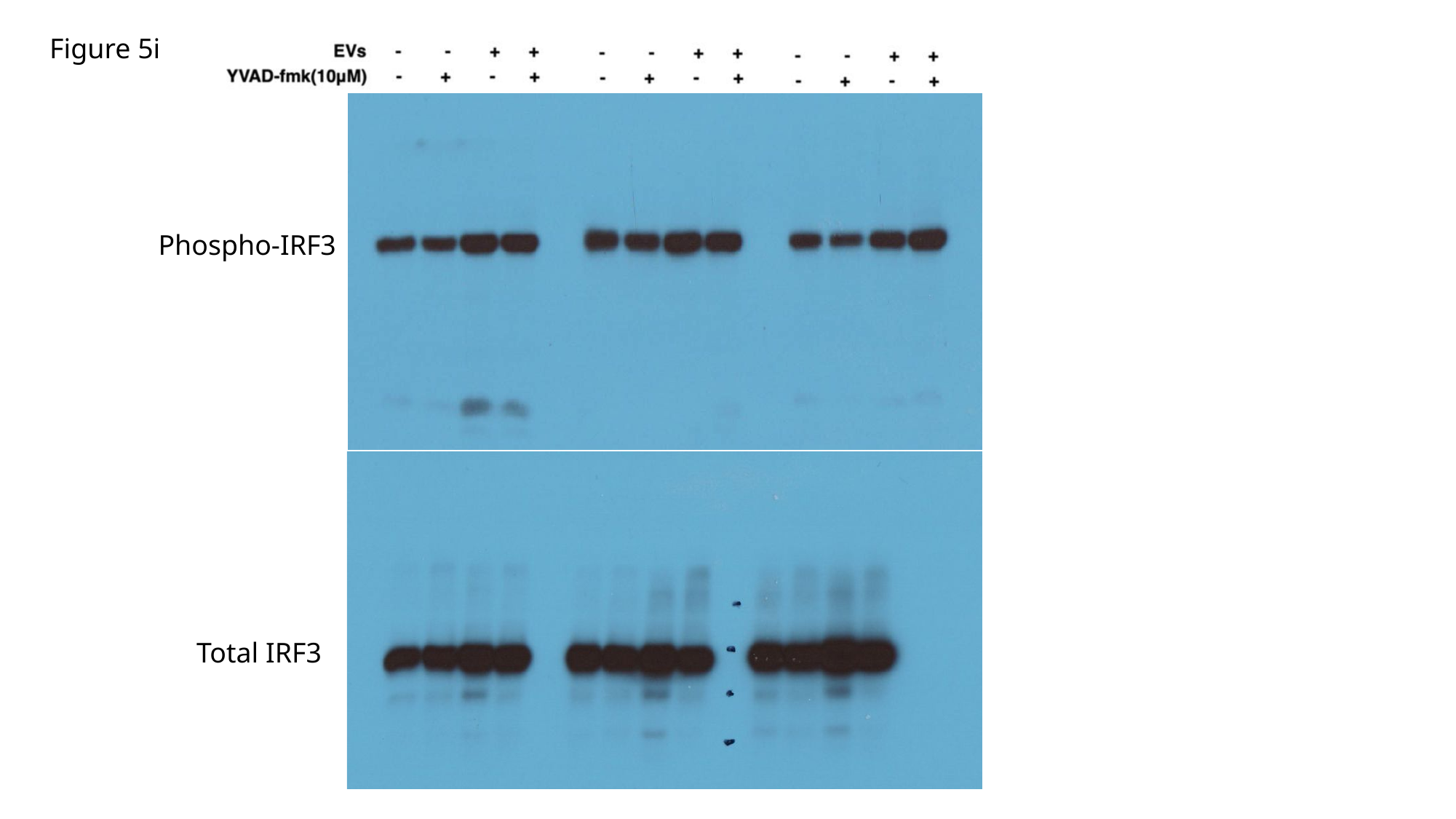

Figure 5i
Phospho-IRF3
Total IRF3

## Slide 9
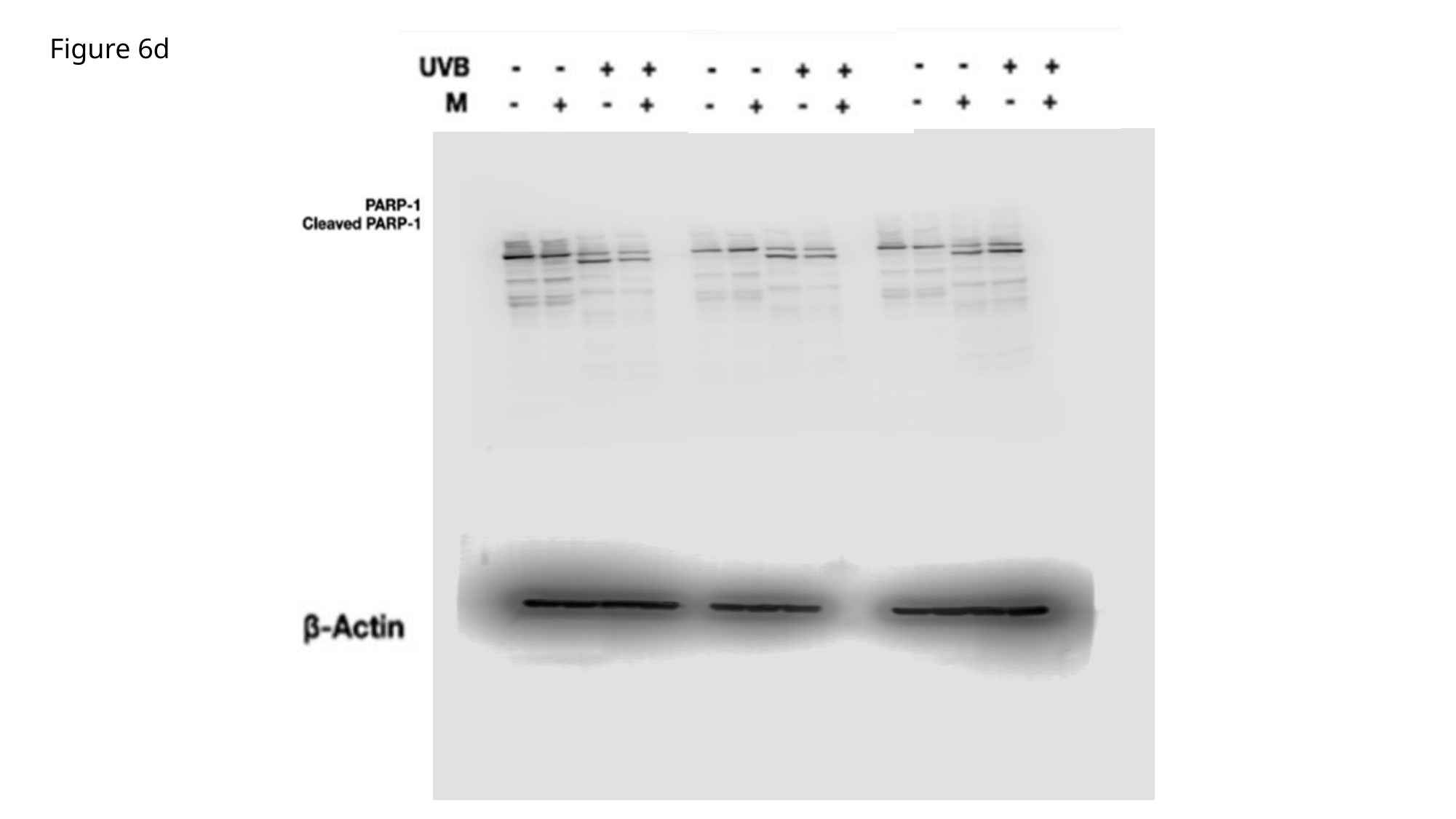

Figure 6d

## Slide 10
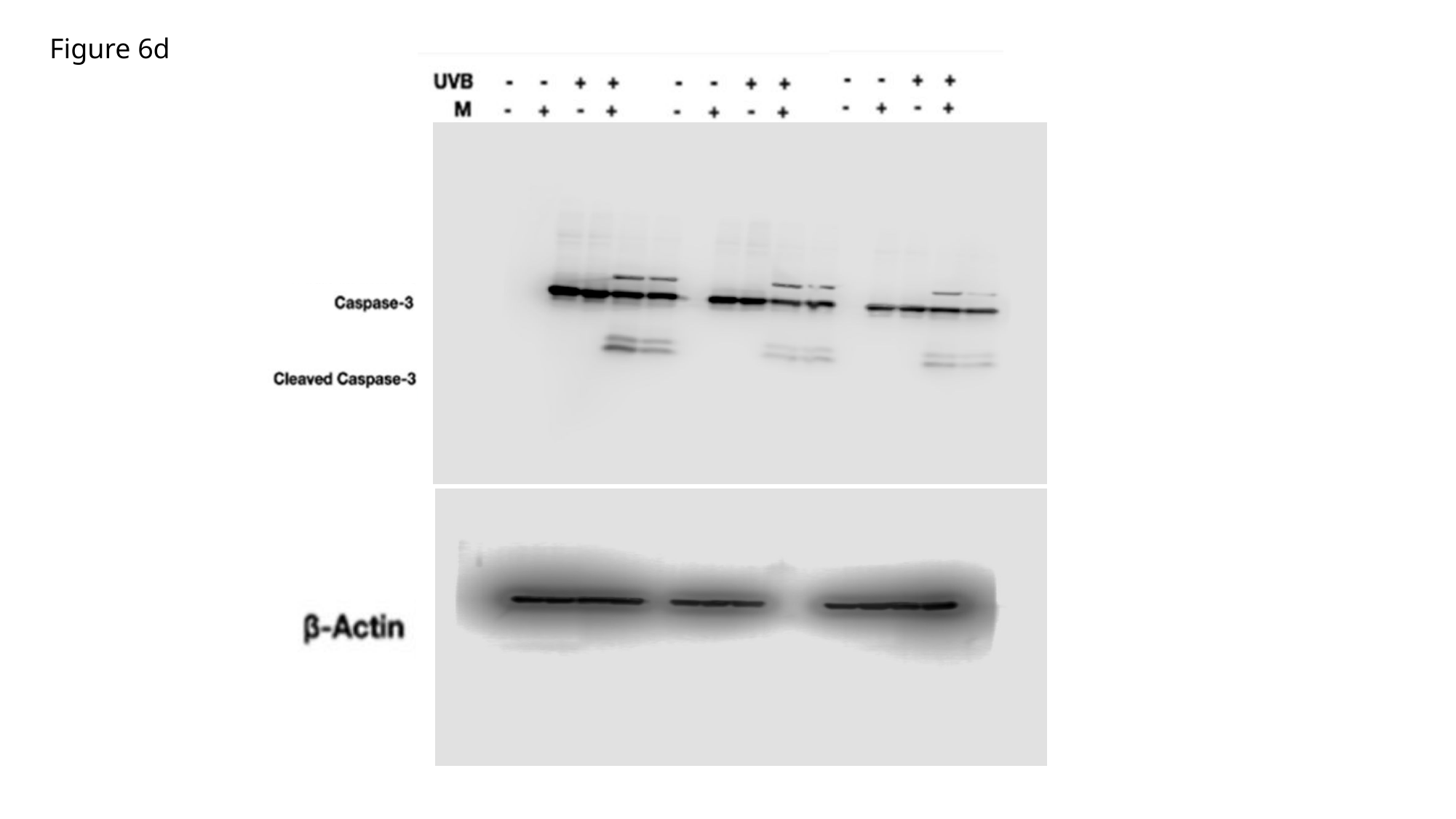

Figure 6d
